# Bamboo-Enabled Nanomaterials for Biomedical Applications

**DOI:** 10.3390/polym18141685

**Published:** 2026-07-08

**Authors:** Hsiuying Wang

**Affiliations:** Institute of Statistics, National Yang Ming Chiao Tung University, Hsinchu 300093, Taiwan; wang@stat.nycu.edu.tw

**Keywords:** bamboo, nanomaterials, biomedical applications, nanocellulose, lignin nanoparticles, silica nanoparticles, carbon quantum dots, carbon-based nanostructures

## Abstract

Bamboo, a fast-growing and sustainable biomass, has traditionally been used in structural applications; however, its hierarchical architecture and rich chemical composition enable both the derivation of advanced nanomaterials and the fabrication of bamboo-assisted nanostructures. Recent studies demonstrate that such bamboo-based nanomaterials, including nanocellulose, lignin nanoparticles, silica nanoparticles, carbon dots, and carbon-based nanostructures, exhibit unique physicochemical properties suitable for biomedical applications. This review provides an overview of bamboo biology and classification, chemical composition, extraction and synthesis of bamboo-derived nanomaterials, and their biomedical applications. Emphasis is placed on their diverse biomedical applications, including drug delivery, tissue engineering and regenerative medicine, wound healing and antimicrobial dressings, cancer therapy, antioxidant and anti-inflammatory applications, and biomedical imaging and biosensing. In addition, emerging approaches that integrate bamboo-derived materials with plant-based bioactive compounds, particularly rose-derived phytochemicals, are proposed as promising strategies for achieving synergistic, broad-spectrum antibacterial activity against both Gram-positive and Gram-negative bacteria. Overall, bamboo-based nanomaterials offer a sustainable and versatile platform for next-generation nanomedicine, with significant potential for future biomedical innovations.

## 1. Introduction

Bamboo is a fast-growing and highly productive woody plant that adapts well to diverse environmental conditions. Due to its rapid growth, renewability, and economic value, bamboo is often described as “Green Gold” and is categorized as a non-timber forest product with substantial ecological and economic significance [1]. It thrives in diverse soil types, ranging from nutrient-poor to mineral-rich environments, and exhibits strong tolerance to environmental stresses, including drought, waterlogging, and flooding [2].

Bamboo forests possess a strong carbon sequestration capacity and play a significant role in climate change mitigation and global carbon cycling. In addition, bamboo-based products generally exhibit relatively low carbon footprints [3]. However, the carbon footprints of bamboo products vary considerably, with bamboo handicrafts exhibiting the highest average emissions and bamboo construction panels showing the lowest [4]. Compared with other lignocellulosic biomass resources commonly used for nanomaterial production, such as wood and agricultural residues, bamboo offers several advantages, including rapid growth rates, short harvesting cycles, high biomass productivity, and the ability to regenerate without replanting [5]. Bamboo can reach maturity within approximately 3–5 years [6], whereas many hardwood species require 20–50 years to reach harvestable age. These characteristics make bamboo a highly renewable and sustainable feedstock for the production of value-added nanomaterials. Therefore, bamboo-based materials can contribute to achieving the Sustainable Development Goals (SDGs) through both carbon sequestration and the development of environmentally friendly bio-based products.

In terms of mechanical performance, bamboo exhibits excellent mechanical properties, including high strength, good elasticity, stable performance, and low density. These outstanding properties originate from the hierarchical organization of cellulose, hemicellulose, and lignin within the bamboo cell wall, making bamboo a naturally occurring high-performance lignocellulosic polymer composite. Owing to these characteristics, its specific strength and stiffness can surpass those of conventional construction materials such as wood and even steel [7]. Consequently, bamboo is particularly well-suited for use in large-scale architectural and artistic installations.

Despite these advantages, the macroscale application of bamboo is constrained by limitations such as moisture susceptibility, flammability, and long-term durability concerns compared to mineral-based materials like concrete. These limitations restrict its use in high-rise structural systems. To address these challenges, engineered bamboo and composite systems have been developed, including integration with concrete and other materials to enhance mechanical performance and durability [8,9,10]. In addition, advanced processing techniques, such as microwave-assisted delignification, have been shown to significantly improve the mechanical properties of bamboo, enabling performance that rivals or exceeds conventional structural materials [11].

**Figure 1:**
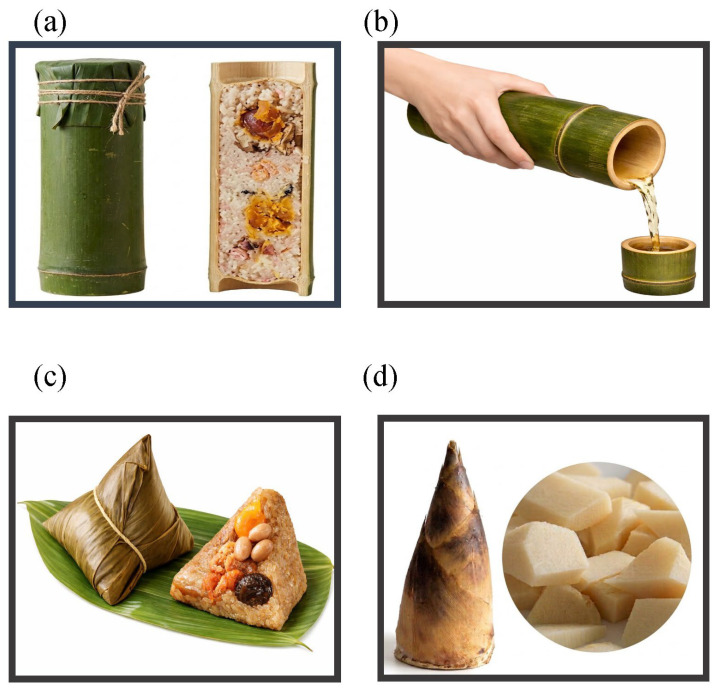
Food-related applications of bamboo: (**a**) bamboo tube rice prepared by cooking rice and ingredients inside bamboo; (**b**) bamboo-infused beverage stored in bamboo tubes; (**c**) sticky rice dumplings wrapped with bamboo leaves; (**d**) young bamboo shoots consumed as a nutritious vegetable.

Beyond construction, bamboo has long been utilized in food-related applications across many Asian countries and other areas. Bamboo tubes are traditionally used to prepare bamboo tube rice, in which rice, meat, and vegetables are placed inside the bamboo and cooked, allowing the rice to absorb the natural aroma of the bamboo [12]. Bamboo tubes are also used for storing liquor to produce bamboo-infused beverages [13,14]. In addition, bamboo leaves serve as natural wrappers for sticky rice dumplings, and young bamboo shoots are widely consumed as a nutritious vegetable rich in proteins, carbohydrates, minerals, and dietary fiber, while being low in fat and sugars [15,16]. These food-related applications highlight the versatility and sustainability of bamboo as a natural resource (Figure 1). Interestingly, such traditional uses have also inspired modern nanotechnology strategies; for example, biomimetic encapsulation concepts derived from bamboo leaf wrapping have been applied to stabilize MnO_2_ nanoparticles (NPs) within conductive polymer nanowires, enhancing structural stability and electrochemical performance in flexible fiber electrodes [17].

Beyond these conventional uses, increasing attention has been directed toward engineering bamboo at the nanoscale [18], where nanotechnology offers powerful approaches to tailor its physicochemical and biological properties. Such nanoscale modifications have been shown to enhance performance characteristics, including resistance to microbial degradation and environmental stress [19,20]. In addition, nanotechnology enhances the functionality of bamboo-derived nanomaterials, enabling antimicrobial activity, improved biocompatibility, and interactions with biological systems at the cellular and molecular levels and other applications [21,22]. This review provides a comprehensive overview of bamboo-enabled nanomaterials, focusing on their extraction strategies, structural characteristics, and emerging biomedical applications, including wound healing and antimicrobial dressings, drug delivery, tissue engineering and regenerative medicine, biomedical imaging and biosensing, cancer therapy, antioxidant, and anti-inflammatory systems, as well as implant coatings and biomaterial reinforcement.

## 2. Botanical Classification and Structural Characteristics of Bamboo

Bamboo is a member of the grass family (Poaceae) and is classified within the subfamily Bambusoideae [23]. This subfamily is the only lineage within Poaceae that includes woody species [24]. Approximately 1500 bamboo species have been identified and are classified into three tribes: Bambuseae (tropical woody bamboos), Arundinarieae (temperate woody bamboos), and Olyreae (herbaceous bamboos) [25]. While Bambuseae and Olyreae are closely related as sister groups, Arundinarieae are evolutionarily distinct as temperate specialists [26]. The primary difference lies in their adaptation to different climatic regimes [27].

**Figure 2:**
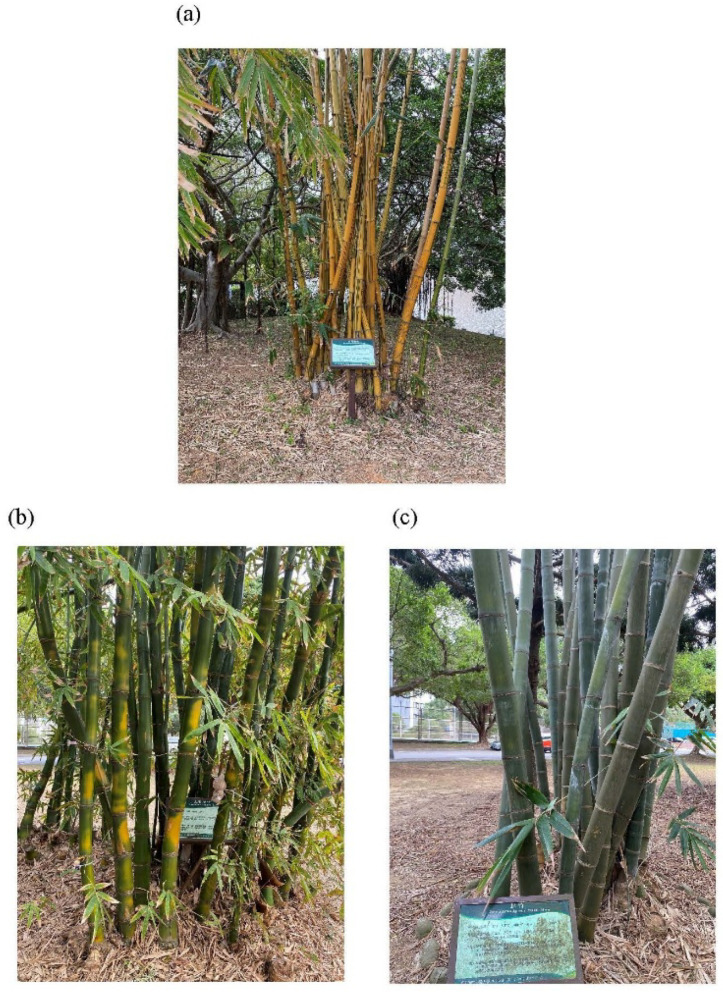
Representative bamboo species (Bambuseae tribe): (**a**) *Dendrocalamus latiflorus*; (**b**) *Bambusa edulis*; and (**c**) *Dendrocalamus giganteus*.

**Figure 3:**
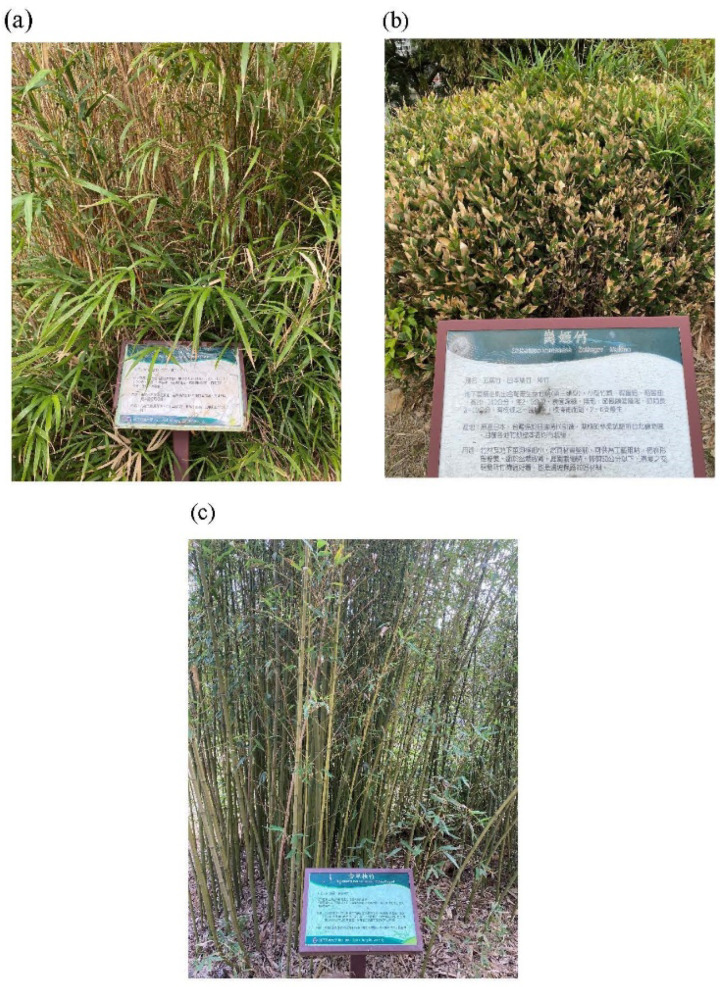
Representative bamboo species (Arundinarieae tribe): (**a**) *Pleioblastus simonii*; (**b**) *Shibataea kumasasa*; and (**c**) *Phyllostachys makinoi*.

Bambuseae represents a monophyletic tribe of tropical woody bamboos, typically characterized by sympodial (clumping) rhizome systems (Figure 2) [28]. Arundinarieae are a tribe of temperate-adapted, woody bamboos, primarily characterized by leptomorph (running) rhizomes that enable aggressive horizontal spreading (Figure 3) [29]. Olyreae is herbaceous with reduced woody structures.

**Figure 4:**
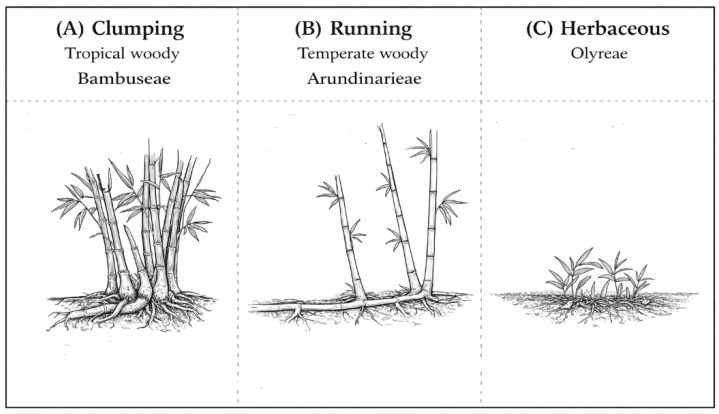
Schematic comparison of the three major bamboo growth forms: (**A**) clumping (Bambuseae) with sympodial rhizomes and clustered woody culms; (**B**) running (Arundinarieae) with leptomorph rhizomes and horizontally spreading culms; and (**C**) herbaceous bamboos (Olyreae) characterized by non-woody culms and understory growth habit.

Figure 4 shows the rhizome growth patterns of the three tribes.

Among the three major bamboo lineages within Bambusoideae, nanomaterial extraction has predominantly focused on woody bamboos belonging to Bambuseae and Arundinarieae, owing to their high cellulose content, dense vascular bundle architecture, and superior crystallinity [30,31]. Although herbaceous bamboos (Olyreae tribe) also contain cellulose, their lower degree of lignification and reduced fiber density have limited their exploration as precursors for high-performance nanomaterials. Nevertheless, non-woody biomass can also serve as a viable source of nanocellulose particles [32].

**Figure 5:**
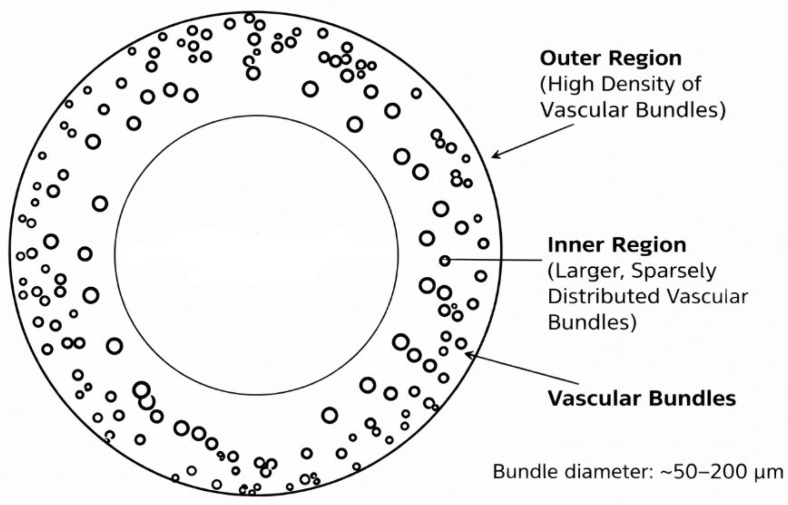
Schematic cross-sectional anatomy of bamboo culm. The vascular bundles exhibit a radially graded distribution, with a higher density of smaller bundles in the outer region and larger, more sparsely distributed bundles toward the inner region.

From an anatomical perspective, bamboo is an anisotropic and functionally graded material characterized by an atactostele-like microchannel system. This structure consists of vascular bundles, comprising metaxylem, protoxylem, and phloem, encased by lignocellulosic sclerenchyma fibers and embedded within a matrix of living parenchymal tissue. The vascular vessels are radially distributed from the inner to the outer wall of the internode culm, with diameters typically ranging from 50 to 200 µm [33]. The size of vascular bundles in bamboo exhibits a radial gradient, with smaller and more densely distributed bundles in the outer region, and larger, more sparsely distributed bundles toward the inner region [34] (Figure 5).

**Figure 6:**
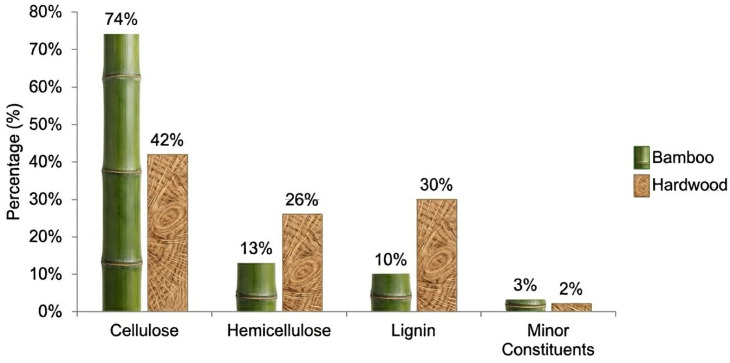
Chemical composition of bamboo fibers and hardwood. A comparative analysis of the primary and secondary constituents of extracted bamboo structural fibers and traditional hardwood, illustrating the significantly higher cellulose content and lower lignin levels inherent to bamboo’s reinforcing fiber bundles. Values for minor constituents are derived from average reported data for wood and bamboo.

At the nanoscale, bamboo exhibits a two-phase structure in which cellulose acts as the reinforcing component, embedded within a matrix of lignin and hemicellulose [35]. While the overall composition of bamboo typically ranges from 40–55% cellulose, 5–30% hemicellulose, and 20–30% lignin, extracted structural bamboo fibers have been reported to contain approximately 74% cellulose, 13% hemicellulose, and 10% lignin [36]. In comparison, typical hardwood tissues contain approximately 40–50% cellulose, 15–35% hemicellulose, and 18–25% lignin (Figure 6).

Despite the broadly similar chemical composition of bamboo, different species exhibit significant variations in cellulose, hemicellulose, lignin, and other minor constituents. These compositional differences can affect the production, yield, and properties of bamboo-derived nanomaterials [37]. Species-specific variations in chemical composition may influence nanomaterial morphology, physicochemical properties, and biomedical performance, including mechanical reinforcement, drug-loading capacity, biodegradability, and biocompatibility. Therefore, consideration of bamboo species is important for the rational selection of feedstocks for specific nanomaterial production and biomedical applications.

## 3. Nanotechnology

Bamboo has emerged as a versatile and sustainable precursor for the development of diverse nanomaterials. Bamboo-enabled nanomaterials encompass a wide range of functional systems, including nanocellulose, lignin NPs (LNPs), silica NPs (SiO_2_ NPs), carbon quantum dots (CQDs), carbon-based nanostructures, as well as green-synthesized metal and metal oxide NPs. These nanomaterials can be obtained through various extraction and synthesis strategies, primarily involving mechanical, chemical, and enzymatic approaches.

### 3.1. Extraction Method

The nanomaterial extraction strategies applied to bamboo are largely adapted from established lignocellulosic nanocellulose processing routes [38], including chemical, mechanical, enzymatic, and oxidative treatments. However, due to bamboo’s dense vascular bundle architecture, high silica content, and elevated crystallinity, processing conditions often require modification compared with conventional wood-based systems. Prior to the production of nanocellulose, cellulose is typically isolated from bamboo through a series of pretreatment and purification steps designed to remove lignin, hemicellulose, extractives, and other non-cellulosic components.

The major challenge in bamboo-derived nanocellulose production is achieving scalable, cost-effective isolation with consistent quality, alongside the need for standardized characterization protocols. Nanocellulose extraction methods can generally be classified into mechanical, chemical, and biological approaches. These techniques may also be combined to optimize product quality, yield, and environmental sustainability [39]. Beyond extraction, functionalization further enhances performance and expands applicability. Chemical modifications (e.g., 2,2,6,6-tetramethylpiperidine-1-oxyl (TEMPO) oxidation, silanization) improve surface reactivity and stability, physical approaches (e.g., blending, coating) enhance compatibility, and biological conjugation imparts bioactivity [40]. These methods enable the conversion of raw bamboo, a sustainable biomass resource, into high-performance reinforcing fillers for polymer composites.

Mechanochemical approaches have recently attracted considerable interest in the development of polymer nanocomposites due to their effectiveness, environmental sustainability, scalability, and simplicity. The most commonly employed mechanical techniques include milling, ultrasonication, and high-shear mixing in the development of polymer nanocomposites [41]. In the milling method, nanoparticle aggregates are ground between small beads in a rotating chamber [42]. This ultrasonication technique employs high-frequency sound waves to generate cavitation bubbles in liquid dispersions [43]. The collapse of these bubbles produces intense local pressure and shear forces that help disperse and break apart nanoparticle aggregates. High-shear homogenization is a simple and cost-effective technique for producing nanocellulose. This method utilizes rotor–stator mixers consisting of a stationary stator and a rapidly rotating rotor that generates strong shear forces [44]. These forces break down cellulose fibers into nanoscale fibrils, facilitating the formation of nanocellulose.

The chemical approach includes acid hydrolysis and oxidation (e.g., TEMPO). Acid hydrolysis is one of the most widely used chemical methods for producing nanocellulose [45], particularly cellulose nanocrystals (CNCs) [46]. In this process, strong acids such as sulfuric acid or hydrochloric acid selectively hydrolyze the amorphous regions of cellulose fibers while preserving the highly ordered crystalline domains. The removal of amorphous components results in rod-like or spherical nanocellulose particles with high crystallinity, large surface area, and good mechanical properties. Oxidation is an important chemical method for producing nanocellulose [47]. For example, TEMPO-mediated oxidation converts surface hydroxyl groups into carboxyl groups, increasing electrostatic repulsion between fibrils. This weakens hydrogen bonding and facilitates the mechanical disintegration of cellulose into nanofibrils or microfibrillated cellulose.

Conventional chemical and mechanical methods for nanocellulose production are energy-intensive, environmentally harmful, and may cause component loss. The enzymatic method is a green approach for producing nanocellulose from lignocellulosic biomass such as bamboo [48]. Compared with chemical methods, this approach operates under mild conditions and reduces chemical consumption and environmental impact. Enzymes, mainly cellulases and hemicellulases, selectively hydrolyze hemicellulose and the amorphous regions of cellulose, weakening the fiber structure and facilitating the separation of cellulose into nanoscale fibrils. Enzymatic pretreatment is usually combined with mechanical processes to improve fibrillation efficiency. A green strategy combining dual-enzymatic treatment and ball milling was developed to produce nanocellulose from bamboo pulp with varied morphologies and structures [49].

The major extraction methods used to produce nanocellulose, including cellulose nanocrystals (CNCs) and cellulose nanofibrils (CNFs) are summarized in Table 1 [46,47,48,49,50,51].

In addition to these individual methods, combinations of approaches can also be employed to produce nanomaterials from bamboo. Bamboo nanocellulose can be prepared using a combined chemical-mechanical extraction process, involving sodium hydroxide (NaOH) pretreatment (alkali treatment) followed by high-speed ball milling [52]. The NaOH treatment reduced lignin content, while mechanical milling generated nanofibrils with particle sizes around 80 nm. Structural and microscopic analyses confirmed changes in crystallinity and morphology. When incorporated into epoxy composites, the nanocellulose improved tensile strength, with optimal performance observed at 1.5 wt% loading, demonstrating its potential as a reinforcing nanomaterial. In addition to bamboo, combined approaches have also been applied to produce nanocellulose from other sources. Chemical pretreatments, including pulping, bleaching, and acid hydrolysis, are used to remove lignin, hemicellulose, and extractives from pineapple leaf fibers, followed by high-shear homogenization and ultrasonication to generate nanocellulose [53].

In bamboo-derived materials, effective pretreatment is essential to overcome intrinsic biomass recalcitrance associated with its highly organized lignocellulosic structure. Nevertheless, traditional methods such as dilute acid, alkaline, and organosolv treatments remain chemically intensive and operationally complex, with persistent challenges in solvent stability and recovery. The development of sustainable and energy-efficient pretreatment technologies is therefore crucial for advancing bamboo-based nanomaterial production [54]. In addition, conventional nanocellulose production methods may present environmental and economic challenges. Recent studies have therefore focused on developing more sustainable production strategies, including the use of renewable biomass sources, reduced consumption of hazardous chemicals, lower energy requirements during mechanical processing, and improved recycling and regeneration of process streams [55].

### 3.2. Nanomaterials

Bamboo serves as a versatile precursor for a wide range of nanomaterials, including nanocellulose, lignin NPs, silica NPs (SiO_2_ NPs), carbon quantum dots (CQDs), and carbon-based nanostructures.

#### 3.2.1. Nanocellulose

Cellulose is the most abundant structural component of plant cell walls and can be obtained from a wide range of sources, including agricultural waste, wood, natural fibers, and other plant-derived materials [56]. Nanocellulose is a nanoscale material obtained from cellulose, a naturally occurring structural polysaccharide found in plants and produced by certain bacteria [57,58]. It has attracted considerable attention owing to its exceptional mechanical properties, high specific surface area, tunable surface chemistry, and intrinsic sustainability [59].

Nanocellulose is generally classified into three principal categories: (1) CNCs, also known as cellulose whiskers; (2) CNFs, sometimes termed nanofibrillated cellulose (NFC) or microfibrillated cellulose (MFC); and (3) bacterial cellulose (BC), which is biosynthesized by specific microorganisms [60]. In addition, electrospun cellulose nanofibers (ECNFs) represent a regenerated cellulose nanostructure produced through electrospinning techniques.

The diameter of nanocellulose commonly ranges from 5 to 100 nm [61]. CNC, CNF, and NFC/MFC can be derived from bamboo. The incorporation of white bamboo NFC into epoxy resin composites was shown to significantly enhance flexural and tensile strength, fracture toughness, thermal stability, and overall stiffness [62]. The production of nanocellulose typically involves the following steps, although variations exist. Raw fibers are first milled and then subjected to alkali and NaClO_2_ bleaching treatments to remove lignin and hemicellulose while preserving the cellulose structure. The resulting bleached fibers are subsequently processed either by acid hydrolysis or by mechanical disintegration under high-pressure shear [63]. Spherical cellulose nanocrystals were extracted from Gai bamboo (*Bambusa blumeana*) fibers using sulfuric acid hydrolysis [64]. Microfibrillated cellulose nanofibers were produced efficiently by using TEMPO oxidation as a pretreatment prior to mechanical processing [48].

#### 3.2.2. Lignin Nanoparticles

Lignin is a natural biopolymer commonly used as a fuel for energy production. Lignin-based NPs have potential applications in functional surface coatings, nanoglue, drug delivery, and microfluidic devices [65]. Lignin NPs exhibit excellent adsorption capacity, biodegradability, and low toxicity, making them suitable carriers for drug molecules and inorganic particles [66]. In addition, lignin NPs are promising biodegradable carriers for biocidal agents with low environmental impact [67].

A coupled catalytic hydrothermal pretreatment–hydrophobic acidic deep eutectic solvent cascade fractionation strategy effectively separated hemicellulose and lignin from *Dendrocalamus latiflorus* bamboo biomass, resulting in high sugar yields and antioxidant-active lignin nanoparticles (~31 nm) [68]. Using a tetrabutylammonium bromide and glyoxylic acid monohydrate deep eutectic solvent, bamboo was selectively fractionated to obtain preserved cellulose and structurally intact lignin; the cellulose was converted into CNFs, and the extracted lignin was further used for lignin NP preparation and alginate-based bioseparation beads [69]. A microwave-assisted deep eutectic solvent pretreatment enabled one-pot fractionation of bamboo into cellulose-rich residues and lignin, which were further converted into lignin-containing cellulose nanofibrils and lignin NPs [70]. The lignin underwent in situ reactions that enhanced its amphiphilicity and facilitated self-assembly for lignin NP preparation.

Supramolecular assembly of lignin into micro- and NPs (LMNPs) has recently gained significant attention. Supramolecular LMNPs were produced from bamboo acetosolv lignin by tuning initial lignin concentration, antisolvent addition rate, temperature, and coexisting chemicals [71]. Controlled conditions yielded diverse morphologies and sizes, providing insights into LMNP formation mechanisms and enabling morphology-tailored nanomaterials for potential applications.

A rapid formic acid-based fractionation of bamboo enabled efficient separation of cellulose and lignin for integrated nanomaterial production [72]. The bamboo-derived cellulose was readily converted into CNCs via TEMPO oxidation, while lignin formed uniform NPs. The resulting CNCs/lignin NP nanocomposite membranes showed smooth morphology, enhanced mechanical properties (44% higher tensile strength and 47% higher Young’s modulus at a 5:1 ratio), and effective antibacterial activity against *Escherichia coli*.

#### 3.2.3. Silica Nanoparticles

Silica NPs are nanoscale materials composed of silicon dioxide, typically exhibiting particle sizes below 100 nm [73] and widely used due to their chemical stability, high surface area, and ease of surface modification. Advanced bioanalysis, including precise quantification, requires understanding biological and medical processes at the molecular level. Bioconjugated silica NPs offer a promising approach to meet this need [74].

Silica NPs synthesized by a water-in-oil microemulsion method can be bioconjugated with biomolecules such as enzymes, antibodies, and DNA, enabling medical applications in biosensing, bioimaging, cell staining, DNA detection, and rapid bacterial identification [75]. Bamboo leaf ash has been identified as a naturally amorphous material primarily composed of silicon dioxide [76]. Silica NPs were synthesized from bamboo leaf waste through thermal combustion and alkaline extraction as a low-cost and sustainable approach [77]. The resulting amorphous NPs (~25 nm) exhibited high surface area and high purity (99%).

#### 3.2.4. Carbon-Based Nanomaterials

Carbon-based nanostructures include several nanomaterials with potential medical applications, such as carbon quantum dots, graphene, and graphene oxide, which can be derived from bamboo.

Carbon quantum dots (CQDs) are an emerging class of fluorescent carbon nanomaterials that have attracted considerable attention as alternatives to conventional semiconductor quantum dots [78]. CQDs exhibit excellent biocompatibility, strong catalytic activity, and unique optical properties, making them promising materials for luminescent devices and applications in biomedical sensing, optical, and photonic technologies [79]. Organosolv lignin extracted from Moso bamboo waste was used as the carbon source to synthesize CQDs through a simple two-step method [80]. 2,4-Diaminobenzenesulfonic acid served as the nitrogen and sulfur dopant. After hydrothermal treatment at 200 °C for 12 h, the resulting N, S-doped CQDs exhibited strong green fluorescence with a maximum quantum yield of 17.7%. These CQDs also showed selective fluorescence quenching toward Fe^3+^, indicating their potential as fluorescent probes for metal ion detection.

Graphene is a single layer of sp^2^-hybridized carbon atoms arranged in a tightly packed two-dimensional honeycomb lattice [81]. Owing to its outstanding electronic, physical, chemical, and mechanical properties, graphene has attracted enormous scientific interest. Graphene-like materials were synthesized from bamboo waste through pyrolysis followed by solvent treatments [82]. Characterization confirmed the formation of carbon-rich structures with good electron transfer and enhanced current response compared with charcoal-derived materials. A bio-based graphene material was synthesized from ethanosolv lignin derived from bamboo via iron-catalyzed graphitization, followed by the improved Hummers’ method and low-temperature thermal annealing [83]. During graphitization, lignin was converted into turbostratic crystallites with a flake-like structure. After oxidation, graphene layers were exfoliated and their interlayer spacing increased, while the lamellar morphology was largely preserved after reduction. The graphitized lignin showed high carbon content and low oxygen content, whereas graphene oxide exhibited higher oxygen content due to oxidation.

Compared with graphene, graphene oxide has advantages such as lower production cost, scalability, and easier processing, and is commonly used as a precursor for reduced graphene oxide [84]. An eco-friendly green synthesis of reduced graphene oxide was reported using bamboo shoot extract as a natural reducing agent [85]. The obtained bamboo-derived reduced graphene oxide was confirmed to have removed oxygen-containing groups and formed reduced graphene oxide. The material exhibited ultrathin sheet-like structures and showed high electrochemical performance with a specific capacitance of 514.6 F/g, indicating its potential application in supercapacitors.

#### 3.2.5. Carbon-Based Nanostructures

Some carbon nanostructures have emerged as fluorescent materials with significant potential in optoelectronics, chemical sensing, bioimaging, and energy storage applications [86]. Bamboo can be converted into carbon-based materials such as charcoal and biochar, which possess hierarchical micro-/nano-porous structures. Bamboo charcoal is a porous carbon material with a hierarchical micro-/nano-porous structure characterized by small pore size and low pore volume [87,88], and such porous carbon materials can serve as precursors for the synthesis of carbon-based nanomaterials.

**Figure 7:**
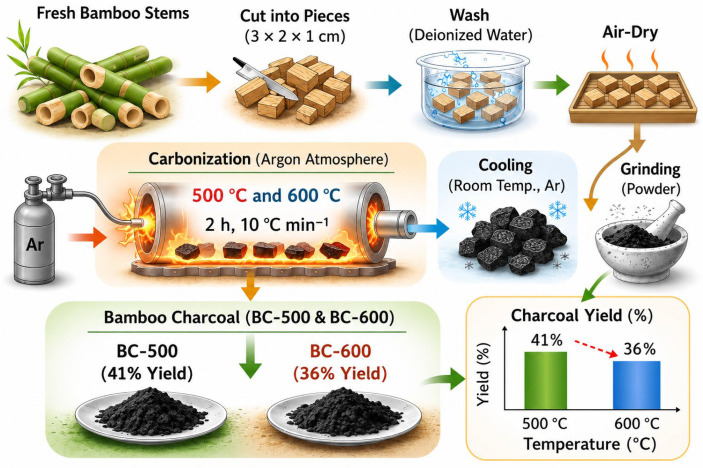
Schematic illustration of the preparation of bamboo charcoal through cutting, washing, drying, carbonization, cooling, and grinding.

The production process of bamboo charcoal is as follows [86]. Fresh bamboo stems (*Bambusa vulgaris*) were cut into small pieces, washed with deionized water, and air-dried. The dried samples were carbonized at 500 and 600 °C for 2 h under an argon atmosphere with a heating rate of 10 °C min^−1^, followed by cooling to room temperature under inert conditions. The resulting carbonized materials were ground into powder to obtain bamboo charcoal, denoted as BC-500 and BC-600 according to the carbonization temperature. The charcoal yield was calculated based on the weight ratio of the final product to the raw bamboo. The yield decreased from approximately 41% at 500 °C to 36% at 600 °C, indicating reduced carbonization yield at higher temperatures (Figure 7).

Bamboo charcoal is a form of carbon with a hierarchical micro-/nano-porous structure characterized by small pore size and low pore volume [87,88], and such porous carbon materials can serve as precursors for the synthesis of carbon-based nanomaterials. In mountainous bamboo-growing areas, converting bamboo waste into bamboo charcoal using charcoal kilns has become a widely adopted practice due to the simplicity of kiln construction and operation [89].

Bamboo-derived biochar and charcoal are both amorphous forms of black carbon with abundant pores and surface irregularities [90]. Bamboo biochar generally exhibits higher porosity and a larger specific surface area than bamboo charcoal, with even small amounts possessing extremely high internal surface areas. This porous structure enhances soil properties by improving water retention, nutrient adsorption, and providing habitats for beneficial microorganisms. In contrast, bamboo charcoal typically shows lower porosity and surface area due to differences in processing conditions.

## 4. Medical Applications and Physicochemical Properties

### 4.1. Medical Applications

**Figure 8:**
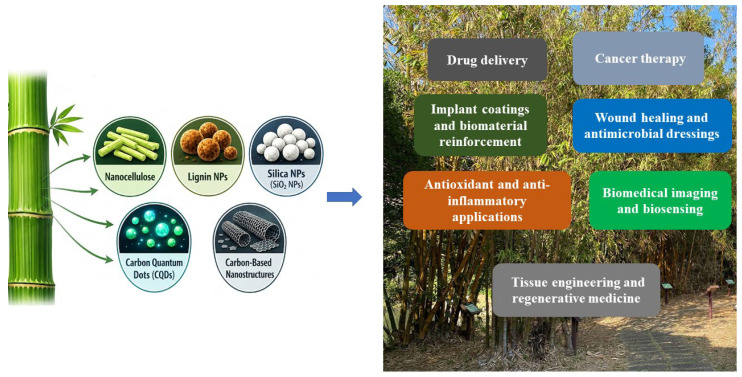
The diverse biomedical applications of bamboo-derived nanomaterials.

Bamboo-based biomaterials and bamboo-assisted nanocomposites exhibit a wide range of medical applications, including wound healing and antimicrobial dressings, drug delivery, tissue engineering and regenerative medicine, biomedical imaging and biosensing, cancer therapy, antioxidant and anti-inflammatory applications, and biomaterial reinforcement (Figure 8).

#### 4.1.1. Wound Healing and Antimicrobial Dressings

Wound healing is a natural biological process in the human body that proceeds through four well-coordinated and highly regulated phases: hemostasis, inflammation, proliferation, and remodeling. Successful healing requires that these phases occur in the correct sequence and within an appropriate time frame. However, various factors may disrupt one or more stages, leading to delayed or impaired wound healing [91]. Therefore, effective wound management is critically important in clinical practice. Chronic and acute wounds pose significant clinical challenges, particularly due to infection risk, which can delay healing and increase morbidity. Antimicrobial dressings and nanomaterials have emerged as an effective strategy to control microbial burden and promote wound healing [92,93].

Silver nanoparticle-impregnated bamboo cellulose nanocrystal nanobiocomposites were evaluated for diabetic wound healing in streptozotocin-induced mice [22]. Topical hydrogel treatment achieved nearly complete wound closure (98–100%) within 18 days, compared with 88–92% in control groups. The treatment reduced pro-inflammatory cytokines and enhanced collagen and growth factor expression. These results suggest that bamboo-based nanobiocomposites may serve as promising biocompatible wound-healing dressings.

Silver NPs synthesized via bamboo leaf extract have been demonstrated to accelerate burn wound healing through enhanced antimicrobial activity and promotion of tissue regeneration [94]. Bamboo-derived silver NPs exhibit strong antibacterial activity against both Gram-positive and Gram-negative pathogenic microorganisms, along with notable antioxidant activity against 2,2-diphenyl-1-picrylhydrazyl (DPPH) free radicals [95]. A simple two-step surface modification was used to create stable silver nanoparticle coatings on bamboo. L-3,4-dihydroxyphenylalanine (L-DOPA) formed a catechol-rich layer that reduced and anchored silver NPs, while polyethyleneimine (PEI) further increased silver loading and coating stability. The modified bamboo became more hydrophobic and showed strong antimicrobial performance, achieving complete inhibition of *Escherichia coli* and *Staphylococcus aureus*, as well as improved antifungal activity against Aspergillus niger. This approach offers a practical way to produce antimicrobial bamboo materials for sustainable applications [96].

#### 4.1.2. Anticancer Activity

Cancer remains a major global health challenge, and conventional therapies are often limited by toxicity, drug resistance, and poor targeting. Nanomaterials offer promising alternatives due to their unique properties and have attracted attention for anticancer applications because of their biocompatibility, sustainability, and multifunctionality [97,98].

Plant-mediated biosynthesis of silver NPs has emerged as a promising alternative to conventional chemical synthesis methods. Silver NPs synthesized from the leaves of Bambusa arundinacea and Bambusa nutans have been evaluated for their anticancer activity against human prostate cancer cell lines [99]. The results indicated that bamboo-derived silver NPs exhibit potential anticancer activity against PC3 cells. Silver NPs synthesized using bamboo leaf extracts have demonstrated additional bioactivities, including antioxidant and anticancer effects [95].

NPs have been increasingly explored as nanomedicine for the effective treatment of diabetes. In particular, zinc oxide NPs synthesized using Bambusa arundinacea have demonstrated significant biological activities [100]. Histopathological studies conducted on male Wistar rats confirmed their therapeutic potential. The biosynthesized zinc oxide NPs exhibited notable antihyperglycemic effects, along with strong antibacterial activity against pathogenic microorganisms and promising anticancer properties. d-α-Tocopherol polyethylene glycol 1000 succinate (TPGS)-functionalized bamboo charcoal NPs loaded with curcumin demonstrated enhanced anticancer efficacy through combined chemotherapy and near-infrared (NIR)-induced photothermal therapy [101]. This synergistic approach improved therapeutic outcomes both in vitro and in vivo. Additionally, the composite exhibited free radical-scavenging activity. Enhanced hot-water extracts of Kumaizasa bamboo leaves exhibited significant immunostimulatory and antitumor activities by activating natural killer cells and macrophages, increasing cytokine production, and suppressing tumor growth in mouse models [102]. These effects were mainly attributed to a 1,3-β-glucan-rich fraction and warrant further clinical investigation.

#### 4.1.3. Drug Delivery

Drug delivery plays a crucial role in improving the efficacy and safety of therapeutic agents, particularly in the treatment of complex diseases such as cancer [103]. Conventional delivery systems often suffer from poor bioavailability, rapid degradation, and limited targeting ability. Recent drug delivery systems offer significant advantages over conventional approaches, owing to their improved performance, precision, and therapeutic efficacy. These systems are typically based on nanomaterials or miniaturized devices with multifunctional features, exhibiting properties such as biocompatibility, biodegradability, high viscoelasticity, and prolonged circulation time in the body [104,105].

A sodium alginate-bacterial cellulose nanocomposite hydrogel with multilayer porous structures was fabricated via in situ biosynthesis using enzymatic hydrolysate of glycerol-pretreated Moso bamboo as the carbon source [106]. Sodium alginate was incorporated into bacterial cellulose through hydrogen bonding (0.25–1%), with 0.75% sodium alginate showing improved thermal stability. The hydrogel exhibited pH-responsive swelling behavior and effective drug-release properties, achieving a lysozyme release rate of 92.79% at pH 7.4 over 60 h.

Nano bamboo charcoal, produced from bamboo, was investigated as a sustained drug-release carrier for Eucommia ulmoides extract [107]. The material showed high adsorption capacity (462.96 mg/g), a cumulative drug release of 70.67%, and demonstrated anticancer activity against HCT116 colon cancer cells, indicating its potential as a nano-drug delivery system for traditional Chinese medicine. TPGS-functionalized bamboo charcoal NPs were developed as a nanocarrier system to improve the bioavailability of poorly soluble curcumin [101]. The platform enhances cellular uptake via nanoscale size effects, enables NIR-triggered controlled drug release, and reduces drug efflux through P-glycoprotein inhibition.

Bamboo-derived hydrophobic lignin-containing cellulose nanofibers (H-LCNFs) have been developed to stabilize aqueous two-phase system Pickering emulsions for drug delivery [108]. The H-LCNFs were prepared from bamboo using deep eutectic solvent pretreatment followed by hydrophobization. The resulting water-in-water emulsions exhibited good stability and pH responsiveness and showed high encapsulation efficiency and retention of the hydrophobic compound resveratrol, suggesting potential for drug delivery applications.

Bamboo leaf flavonoids (BLF) suffer from low bioavailability, which can be effectively improved using alginate–chitosan-coated nanoliposomes [109]. These nanocarriers (∼150–230 nm) exhibit high encapsulation efficiency (up to ~83%), enhanced controlled release, and stronger antioxidant activity compared to free BLF. In addition, they significantly increase skin permeability (up to ~2.9-fold) and improve anti-senescence effects in cell models. This delivery system demonstrates strong potential for enhancing the stability, absorption, and bioactivity of BLF in functional food and cosmetic applications.

#### 4.1.4. Tissue Engineering and Regenerative Medicine

Over the past two decades, tissue engineering and regenerative medicine have focused on repairing damaged tissues. Three-dimensional scaffolds and hydrogels, alone or combined with bioactive components, support tissue formation and provide mechanical stability during implantation [110]. Nature-based biomaterials have been increasingly incorporated into tissue engineering and regenerative medicine applications [111,112,113].

A composite scaffold composed of bamboo fiber, nano-hydroxyapatite (n-HA), and poly(lactic-co-glycolic acid) (PLGA) was fabricated using a freeze-drying method [114]. The porous morphology, porosity, and compressive properties of the scaffolds were evaluated under different preparation conditions. The results showed that the incorporation of bamboo fiber improved the structural and mechanical properties of the n-HA/PLGA composite scaffolds and enhanced cytocompatibility, indicating potential applications in bone tissue engineering.

A multifunctional nano-hydroxyapatite-chitosan scaffold reinforced with bamboo and bioactive compounds was developed for osteoarthritis treatment [115]. The scaffold showed good porosity, mechanical strength, cytocompatibility, osteogenic activity, and controlled drug release, indicating its potential for bone repair and cartilage regeneration. In this composite system, nano-hydroxyapatite contributes to osteogenic activity, while bamboo enhances the mechanical properties.

Bamboo-derived scaffolds preserve a hierarchical porous structure and aligned fibers that mimic natural bone, providing mechanical support and guiding cell growth [116]. After functionalization with collagen and chitosan, these nanostructured materials enhance cell adhesion, osteoconductivity, and bone regeneration. Carboxylated bamboo fiber was incorporated into a nano-hydroxyapatite/chitosan scaffold, forming an oriented porous structure via ionic crosslinking [117]. The composite exhibited enhanced compressive strength, high porosity, reduced degradation rate, increased apatite deposition, and improved cell compatibility, suggesting its potential for bone tissue engineering applications.

#### 4.1.5. Biomedical Imaging and Biosensing

Biomedical imaging and biosensing are essential for disease diagnosis and monitoring. Recently, nanomaterials, particularly biocompatible and sustainable ones such as bamboo-derived materials, have shown promise for enhancing imaging performance and sensing sensitivity.

Bamboo serves as an effective natural biotemplate for developing advanced bio-devices and nanomaterials due to its well-aligned, hierarchical microchannel structure, which is difficult to replicate using conventional microfabrication [33]. Its anisotropic architecture enables efficient fluid transport and offers a low-cost alternative to complex fabrication processes. The lignocellulosic matrix can be functionalized at both micro- and nanoscale levels, including the incorporation of plasmonic NPs and conductive nanomaterials, supporting applications in microfluidics, biosensing, electrochemical platforms, and energy devices. Furthermore, carbonized bamboo can yield conductive nanostructured materials, enhancing its potential for sustainable and scalable bio-device applications.

Bamboo contains diverse bioactive phytochemicals with antioxidant and antimicrobial properties that can enhance immunity and support health. These compounds can also be used to synthesize silver NPs by mixing bamboo leaf extract with AgNO_3_ and heating, where natural phytochemicals reduce Ag^+^ into AgNPs [118]. The silver NPs show promising biomedical applications, including antimicrobial activity, wound healing, and biosensing, highlighting bamboo’s potential in nanomedicine and healthcare-related technologies.

Bamboo-derived carbon dots (CDs) provide a low-cost and low-toxicity alternative to conventional fluorescent bioimaging agents. Ethylenediamine doping produces nitrogen-doped carbon dots (N-CDs) with enhanced photoluminescence (quantum yield ~50.9%), ultra-small size (~2.4 nm), and high hydrophilicity [119]. These N-CDs enable effective bioimaging and, when conjugated with folic acid, facilitate targeted imaging of cancer cells.

Bamboo-derived CDs have been developed as sensitive fluorescent probes for detecting the anti-inflammatory drug flufenamic acid [120]. The sensor shows strong binding, fluorescence enhancement, high selectivity, and a low detection limit. It performs reliably in biological samples such as human urine, demonstrating potential for monitoring drug levels in biomedical and environmental applications.

#### 4.1.6. Antioxidant and Anti-Inflammatory Materials

Bamboo leaves and sheaths contain abundant phenolics and flavonoids with strong antioxidant and anti-inflammatory activities. Leaf extracts show higher phenolic content but some cytotoxicity, while sheath extracts effectively reduce oxidative stress and inflammation without harming cells [121]. These results highlight the potential of bamboo by-products for applications in nutraceutical, cosmetic, and pharmaceutical fields [122].

These bioactive components have also been utilized in nanotechnology, particularly in the green synthesis and functionalization of NPs for biomedical applications.

A bamboo-derived carbon dot-methotrexate (MTX/CD) nanocomplex was developed to improve rheumatoid arthritis (RA) therapy by mitigating MTX-induced oxidative stress [123]. The CDs act as nanocarriers and antioxidant agents, reducing intracellular reactive oxygen species levels while preserving MTX’s anti-inflammatory efficacy. The MTX/CD complex inhibited the proliferation of RA fibroblast-like synoviocytes and enhanced therapeutic outcomes.

Bamboo-derived extracts can act as natural stabilizers in NP synthesis, improving stability and bioactivity [124]. While NPs may exhibit strong antioxidant activity, bamboo-modified NPs can enhance anti-inflammatory effects and maintain stability during storage and processing. These findings highlight the potential of bamboo as a sustainable stabilizer for NP-based biomedical applications.

#### 4.1.7. Biomaterial Reinforcement

Implant coatings and biomaterial reinforcement play a key role in improving the performance and longevity of medical implants [125]. Conventional materials often face challenges such as poor integration, infection risk, and mechanical mismatch. Nanomaterial-based modifications, particularly those derived from sustainable sources like bamboo, offer enhanced biocompatibility, antibacterial activity, and mechanical strength, supporting the development of more effective and durable implant systems.

Chitosan-reinforced bamboo composites incorporating nano-bio-silica (1–3 vol%) were developed via a hand lay-up method [126]. Finite element analysis under static and cyclic loading confirmed its suitability for dental implant applications, with stresses remaining below the yield limits of bone.

The water absorption and porosity in chitosan-based composites reinforced with bamboo fiber, hemp fiber, and varying amounts (1–3%) of nano-bio-silica, fabricated via a composite preparation method (e.g., hand lay-up), were investigated [127]. Water absorption tests showed that 1% nano-bio-silica reduced water uptake, whereas higher silica content increased porosity. Hybrid reinforcement improves overall performance, highlighting the need to balance porosity and mechanical strength for optimal implant design.

Carboxylated bamboo fibers have been incorporated into chitosan/nano-hydroxyapatite systems to fabricate ternary composite membranes for guided bone regeneration via ionic crosslinking [128]. The electrostatic interaction between negatively charged bamboo fibers and protonated chitosan forms a three-dimensional polyelectrolyte network, while nano-hydroxyapatite is embedded through hydrogen bonding. This hierarchical structure significantly enhances mechanical properties, with tensile strength improved by over twofold at an optimal composition. In addition, the composite exhibits controlled degradation and promotes apatite deposition in simulated body fluid, indicating excellent bioactivity.

### 4.2. Structure–Property–Function Interrelationships

The biomedical performance of bamboo-derived nanomaterials is fundamentally governed by the interplay between their structural characteristics, physicochemical properties, and biological functions. Understanding these structure–property–function (SPF) relationships is essential for advancing biomass-based nanotechnology, as specific structural features directly influence material properties and ultimately determine their biomedical performance. Establishing clear links between nanoscale architecture and functional outcomes enables a more rational design of nanomaterials. Such an SPF framework provides valuable guidance for the development of bamboo-derived nanomaterials for biomedical and healthcare applications. The major SPF relationships and extract methods of bamboo-derived nanomaterials discussed in this review are summarized in Table 2.

## 5. Advanced Antibacterial Strategies and Future Perspectives

### 5.1. Antibacterial Strategies

Bamboo leaves have been utilized in traditional Chinese medicine for over a millennium, primarily for the treatment of fever and detoxification [129,130]. Various parts of bamboo have been utilized for therapeutic purposes, including the treatment of skin infections, joint pain, bleeding gums, injuries, fever, and other ailments [131]. Numerous studies have further demonstrated that bamboo leaves exhibit diverse biological activities, including free radical scavenging, antioxidant and anti-aging effects, as well as protective roles against cardiovascular diseases [130,132]. In addition to these therapeutic properties, bamboo also exhibits inherent antibacterial properties, particularly on its outer surface, which provide natural resistance against bacteria, insects, fungi, and other biotic agents [133]. Furthermore, the hierarchical nanostructure of bamboo can enhance its antibacterial properties when combined with other materials. A chitosan (CS)–zinc oxide (ZnO)-based hydrophobic associating hydrogel (HAH) was in situ introduced into the micro-/nanoscale pores of bamboo, markedly improving its antimicrobial and antifouling performance [134].

The emergence of infectious diseases poses a serious threat to public health worldwide, and the rising resistance of bacteria to conventional antibiotic therapies has become a major global health concern [135]. Different types of bamboo exhibit varying antibacterial activities against Gram-positive and Gram-negative bacteria. Gram-positive and Gram-negative bacteria are the two major classes of bacteria, distinguished by the Gram staining method, which reflects differences in their cell wall structure [136]. Gram-positive bacteria possess a thick peptidoglycan layer and lack an outer membrane, allowing them to retain the crystal violet stain and appear purple under a microscope. In contrast, Gram-negative bacteria have a thinner peptidoglycan layer and an additional outer membrane, causing them to lose the crystal violet stain and appear pink or red after counterstaining.

Bamboo leaf extracts have been evaluated against both Gram-negative bacteria (*Escherichia coli*, *Pseudomonas aeruginosa*) and Gram-positive bacteria (*Bacillus subtilis*) [137]. Bamboo charcoal can be used as a support material to prepare antibacterial composites by loading metals such as silver. A multifunctional composite was developed by loading Ag-doped TiO_2_ onto bamboo charcoal via a sol–gel method [138]. The resulting material exhibited enhanced antibacterial activity against *Escherichia coli* and *Staphylococcus aureus*, improved photocatalytic degradation of methylene blue, and effective humidity regulation.

However, bamboo-based materials are seldom integrated with other organic bioactive substances to further broaden their antibacterial spectrum. In contrast, rose extracts have demonstrated inhibitory effects against additional pathogens, including *Pseudomonas aeruginosa*, *Bacillus subtilis*, and *Klebsiella pneumoniae*, which is attributed to their rich content of flavonoids, phenolics, and essential oils [139]. Moreover, Rosa damascena petal extracts and rose oil exhibit antimicrobial activity against both fungi and bacteria [140,141]. Among the tested Gram-positive and Gram-negative bacteria, Gram-positive species, including *Staphylococcus aureus*, *Bacillus subtilis*, and *Streptococcus pyogenes*, showed the highest sensitivity, indicating stronger antibacterial efficacy against these strains.

**Figure 9:**
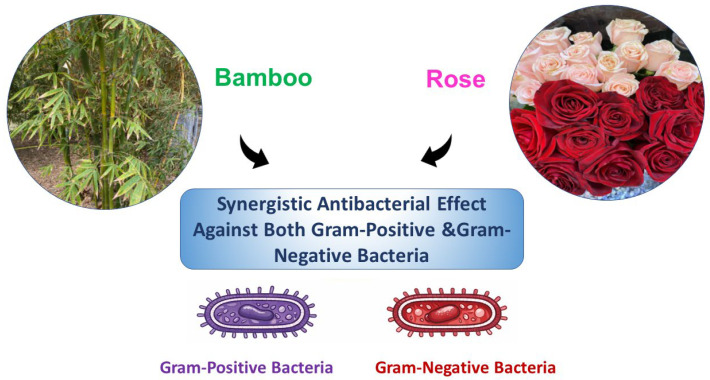
Synergistic antibacterial effect of bamboo-derived materials and rose-based bioactive compounds against both Gram-positive and Gram-negative bacteria.

Owing to these complementary antibacterial spectra, the combination of bamboo-derived materials and rose-based bioactive compounds may potentially provide synergistic effects, which could contribute to broader-spectrum antibacterial activity against both Gram-positive and Gram-negative bacteria (Figure 9). Such integration offers a promising strategy for developing advanced antimicrobial biomaterials with enhanced efficacy.

This work proposes that nanotechnology offers a rational strategy for integrating bamboo-derived structural materials with rose-derived bioactive compounds to develop advanced medical products. Bamboo nanocellulose or nano-structured bamboo carbon can serve as a high-surface-area scaffold, providing mechanical strength, porosity, and biocompatibility. Rose-derived phytochemicals, such as phenolics and essential oil components, can be nano-encapsulated, surface-functionalized, or immobilized within the bamboo matrix to enable controlled and sustained antibacterial release. At the nanoscale, enhanced interfacial interactions increase contact with bacterial membranes, improving antimicrobial efficacy against both Gram-positive and Gram-negative species. Furthermore, nanostructuring improves stability, reduces rapid volatilization of essential oils, and allows tunable release kinetics, which are critical for wound dressings, antimicrobial coatings, and tissue-engineering scaffolds. Thus, nanotechnology not only combines the mechanical advantages of bamboo with the bioactivity of rose but may also enable more precise control over SPF relationships in medical applications. This concept is presented as a future research direction rather than a demonstrated therapeutic strategy, and its feasibility and efficacy require further experimental validation.

### 5.2. Emerging Applications

In addition to construction and nanomedicine applications, bamboo and bamboo-inspired materials have a wide range of industrial applications, including plastics, glass, organic pollutant removal, and scaffolds.

Natural fiber composites have attracted increasing attention due to their environmental sustainability and cost-effectiveness. Among various natural fibers, bamboo stands out for its rapid growth, short cultivation cycle, high strength, and excellent toughness, making it one of the strongest natural fibers available. To address plastic pollution and the recycling challenges of thermosetting plastics, a bamboo-based alternative has been developed [142]. Partial lignin removal and cellulose structure disruption enhanced bamboo plasticity and introduced reactive dialdehyde groups. The activated bamboo was hot-pressed into a moldable thermosetting plastic with tunable color, high solvent and water resistance, strong mechanical properties, and good reusability and biodegradability. Owing to these properties, bamboo-based composites are considered promising alternatives to petroleum-derived plastics across diverse applications [143].

A transparent bamboo composite was developed as a sustainable alternative to conventional glass through lignin modification and epoxy infiltration [144]. The resulting material achieves 87% optical transparency and 118 MPa tensile strength, along with high haze, toughness, low density, and low thermal conductivity, making it promising for energy-efficient building applications.

A Cu_x_O/g-C_3_N_4_-codoped bamboo charcoal composite (Cu-g-C_3_N_4_/BC) was synthesized via in situ pyrolysis for pollutant removal [145]. The Cu-g-C_3_N_4_/BC(600)/H_2_O_2_ system rapidly degraded methylene blue (MB) and rhodamine B (RhB) within 10 min and methyl orange (MO) within 30 min. Synergistic active sites and the conductive bamboo charcoal matrix enhanced adsorption, charge transfer, catalytic efficiency, and reusability for effective organic pollutant degradation.

Decellularized lucky bamboo was demonstrated to be a temporary scaffold for cartilage tissue engineering [146]. It provides high porosity, suitable pore size, and mechanical properties comparable to native cartilage, supporting chondrocyte proliferation, extracellular matrix deposition, and maintenance of the chondrogenic phenotype during culture. These results suggest that bamboo-derived scaffolds may provide structural support during early cartilage regeneration.

A mineralized calcium phosphate/bamboo composite scaffold with a hierarchical porous structure was fabricated using a biotemplating and biomimetic mineralization approach [147]. The scaffold exhibited high mechanical strength with a modulus comparable to cortical bone, along with excellent liquid and cell transport capabilities, including anti-gravity transport. In addition, the composite demonstrated improved osteoconductivity and bone integration due to the synergistic effects of the bamboo template and bioactive mineral phase.

## 6. Limitation and Challenge

Despite their promising biomedical potential, bamboo-derived nanomaterials face several challenges for clinical translation, including long-term biocompatibility, biodistribution, metabolism, and toxicity. Because most current studies remain limited to in vitro or preliminary in vivo animal models, comprehensive in vivo evaluations are still needed to assess pharmacokinetics, biodistribution, therapeutic efficacy, and long-term safety. In addition, variations in bamboo species, growth conditions, harvesting age, and extraction methods may lead to significant batch-to-batch variability in chemical composition, morphology, and physicochemical properties, thereby affecting material reproducibility, quality control, and biological performance [148,149]. Furthermore, the absence of standardized manufacturing processes and regulatory frameworks may hinder the translation of bamboo-derived nanomaterials from laboratory research to clinical applications.

The safety of bamboo-derived nanomaterials, such as CNCs and CNFs, has become a critical topic as these sustainable materials are increasingly used in biomedicine, food packaging, and environmental remediation [150]. Although CNCs and CNFs are sensitive to moisture, hydrophilic in nature, and exhibit relatively low thermal stability [151], they are generally regarded as biocompatible and biodegradable materials [152]. Toxicological evaluations have shown that bamboo leaf antioxidants exhibit low toxicity and no detectable mutagenic effects, despite some reported cytotoxicity at specific concentrations [153]. However, studies investigating the long-term safety, biodistribution, metabolism, and potential chronic toxicological effects of bamboo-derived nanomaterials remain limited [152]. In addition, certain bamboo-derived products may also influence reproductive function, thyroid hormone metabolism, and hepatic drug-metabolizing enzymes [154].

Furthermore, several physicochemical properties of bamboo, such as low solubility and mechanical properties, also limit the applications. Bamboo’s thin-walled and hollow structure can pose challenges for subsequent processing. As a result, appropriate softening pretreatments are often necessary to enable efficient value-added applications [155]. The insolubility of CNCs and CNFs in most common solvents can present challenges during processing and formulation. Their hydrophilic nature results in poor compatibility with many polymer matrices, often causing aggregation, void formation, and weak fiber–matrix interfacial interactions, thereby compromising composite performance [156].

## 7. Conclusions

Bamboo represents a highly sustainable and rapidly renewable biomaterial with remarkable adaptability, positioning it as a key resource in the development of next-generation functional materials. Its intrinsic hierarchical structure, combined with natural antibacterial activity, provides a unique foundation for advanced nanotechnology applications. Recent progress has demonstrated that bamboo-derived nanomaterials, particularly nanocellulose and carbon-based structures, can serve not only as structural scaffolds but also as functional platforms with enhanced physicochemical and biological performance.

Importantly, the integration of bamboo with complementary bioactive components offers a compelling pathway to overcome the limitations of single-material systems. In this regard, this bamboo–rose biomimetic strategy represents a unique cross-disciplinary approach. Combining bamboo-derived nanocellulose with rose-derived phytochemicals introduces synergistic effects, enabling broader-spectrum antibacterial activity and improved functionality. Such hybrid materials have strong potential for applications ranging from biomedical devices to environmental systems, where both antimicrobial efficacy and sustainability are critical.

Looking forward, the rational design of bamboo-based hybrid nanomaterials, guided by biomimetic principles and nanostructural engineering, is expected to play an increasingly important role in addressing global challenges in healthcare and environmental protection. Future research can focus on establishing standardized and scalable production methods, evaluating long-term biosafety and biodegradation, and conducting comprehensive in vivo studies to validate the efficacy of bamboo-derived nanomaterials under clinically relevant conditions. Additional efforts are also needed to investigate pharmacokinetics, biodistribution, immunogenicity, and regulatory considerations to facilitate clinical translation. Such efforts will further expand the potential of bamboo-derived nanomaterials in healthcare.

## Figures and Tables

**Table 1:** Extraction Methods of Bamboo-Derived Nanocellulose.

Category	Method	Principle	Typical Products
Mechanical	Milling	Mechanical size reduction in fibers	CNFs
	Ultrasonication	Acoustic cavitation breaks fiber bundles	CNFs
	High-shear homogenization	Mechanical fibrillation under strong shear forces	CNFs
Chemical	Acid hydrolysis	Removes amorphous regions to obtain crystalline domains	CNCs
	Oxidation (e.g., TEMPO)	Selective oxidation of hydroxyl groups	TEMPO-oxidized CNFs
Enzymatic	Cellulase-assisted hydrolysis	Enzymes selectively degrade amorphous cellulose	CNFs

**Table 2:** Summary of the Structure–Property–Function/Application relationships and extraction methods of the bamboo-derived nanomaterials discussed in this paper.

Material Class	Extraction/Synthesis Method	Structure	Property	Function/Application
Nanocellulose (CNCs/CNFs)	Chemical, mechanical, or enzymatic treatment	High crystallinity, abundant hydroxyl (-OH) groups, rod-like or fibrillar morphology	High mechanical strength, large surface area, tunable surface chemistry, biocompatibility	Tissue engineering, wound healing, drug delivery
Lignin NPs	Solvent extraction and self-assembly	Amorphous polyphenolic structure containing phenolic hydroxyl groups	Antioxidant activity, biodegradability, low toxicity, biocompatibility	Drug delivery, surface coatings, antimicrobial applications
Silica NPs	Chemical extraction from bamboo biomass	Amorphous silica network rich in silanol groups	Biocompatibility, chemical stability, surface functionalization capability	Drug delivery, biosensing, implant reinforcement
Carbon Quantum Dots	Hydrothermal treatment	Carbon nanomaterials with surface functional groups and nitrogen/sulfur doping	Unique optical properties, excellent biocompatibility, strong catalytic activity, selective fluorescence quenching toward Fe^3+^	Fluorescent probes for metal ion detection, biomedical sensing, optical and photonic technologies
Graphene-Based Materials	Pyrolysis, graphitization, oxidation, and reduction	Single layer of sp^2^-hybridized carbon atoms arranged in a two-dimensional honeycomb lattice	Outstanding electronic, physical, chemical, and mechanical properties; high electrochemical performance	Photoelectrochemical devices, supercapacitors
Carbon-Based Nanostructures (Charcoal/Biochar)	Pyrolysis or carbonization	Hierarchical micro-/nano-porous structure; amorphous black carbon with abundant pores and surface irregularities	Electrical conductivity, large surface area, chemical stability	Precursors for carbon-based nanomaterials; chemical sensing, bioimaging, optoelectronics, and energy storage

## Data Availability

No new data were created or analyzed in this study.
